# Effect of Detraining on Muscle Strength, Functional Capacity, Mental Health, and Body Composition in Individuals with Spinal Cord Injury

**DOI:** 10.3390/ijerph21070900

**Published:** 2024-07-10

**Authors:** Lucas Almada, Lucas Santos, Karla Freitas, Joel Rodrigues, Elizângela Diniz, Mauro Mazini-Filho, Luís Leitão, Eveline Pereira, Cláudia Oliveira, Osvaldo Moreira

**Affiliations:** 1 Department of Physical Education, Federal University of Viçosa, Viçosa 36570-900, MG, Brazil; lucas.b.almada@ufv.br (L.A.); lucas.vieira@ufv.br (L.S.); karla.r.freitas@ufv.br (K.F.); joel.a.rodrigues1@gmail.com (J.R.); elizangela.fernandes.f@gmail.com (E.D.); etorres@ufv.br (E.P.); cpatrocinio@ufv.br (C.O.); 2 Southeast Federal Institute of Minas Gerais, Cataguases Campus, Cataguases 36773-563, MG, Brazil; mauro.mazini@ifsudestemg.edu.br; 3 Superior School of Education, Polytechnic Institute of Setubal, 2914-504 Setubal, Portugal; luis.leitao@ese.ips.pt; 4 Institute of Biological Sciences and Health, Federal University of Viçosa, Florestal Campus, Florestal 35690-000, MG, Brazil

**Keywords:** spinal cord injury, muscle strength, functional capacity, mental health, body composition

## Abstract

Spinal cord injury (SCI) is a condition that significantly affects the quality of life (QoL) of individuals, causing motor, physiological, social, and psychological impairments. Physical exercise plays a crucial role in maintaining the health and functional capacity of these individuals, helping to minimize the negative impacts of SCI. The aim of this study was to evaluate the effect of detraining (DT) (reduction or cessation of physical exercise) during the pandemic on five individuals with thoracic SCI. We assessed muscle strength using strength tests, functional capacity using a functional agility test, mental health using anxiety and depression inventories, and body composition using dual-energy X-ray absorptiometry (DEXA). The results after 33 months of DT showed significant losses in functional agility and MS, as well as a worsening in symptoms of anxiety and depression. It was observed that total body mass and fat mass (FM) exhibited varied behaviors among the individuals. Similarly, the results for lean body mass were heterogeneous, with one participant showing significant deterioration. It is concluded that DT caused by the pandemic worsened the physical and mental condition of individuals with SCI, highlighting the importance of continuous exercise for this population and underscoring the need for individual assessments to fully understand the impacts of DT.

## 1. Introduction

Spinal cord injury (SCI) is a debilitating condition characterized by the disruption of neural pathways within the spinal cord, leading to partial or permanent impairment of motor, sensory, and autonomic functions [1,2]. The level and extent of impairment depend on the severity and location of the injury within the spinal cord [3]. This injury can negatively impact an individual’s health and quality of life (QoL), affecting their ability to perform daily activities and fully participate in society [4].

Among the alterations caused by SCI, those impacting health-related variables such as muscle strength (MS), functional capacity (FC), mental health (MH), and body composition (BC) are particularly noteworthy, especially for individuals with SCI [5,6].

BC and MS are essential indicators of health, as their controlled levels and parameters provide metabolic balance and maintain physical mobility, thereby reducing the risk of chronic non-communicable diseases [6,7,8].

FC is an indicator of an individual’s autonomy in performing different daily life activities, ranging from simple to complex, and encompasses physical capacities such as cardiorespiratory fitness, MS, and flexibility, among others, directly influencing an individual’s independence and participation in society [9]. Meanwhile, MH is considered the capacity to connect, function, cope with challenges, and thrive, ranging from optimal well-being to states of intense emotional distress [10]. It influences the individual’s perception of their abilities, coping mechanisms, and overall life satisfaction [11,12]. In the context of SCI, the development and maintenance of these variables are important as they are intrinsically linked to the individual’s rehabilitation and long-term adaptation to the challenges posed by the injury. Therefore, regular physical exercise emerges as an important tool in the development of the aforementioned variables [1]. The human body undergoes acute and chronic physiological adaptations in response to regular physical exercise, enhancing physical and mental health, as well as overall well-being [13]. However, when physical exercise is interrupted or reduced, these adaptations diminish over time, increasing the risk of developing health problems and loss of autonomy and mobility [14,15].

The cessation or reduction of regular physical exercise, known as detraining (DT), negatively impacts the health and physical conditioning of individuals [15,16]. The unique physiological and functional characteristics of individuals with SCI have the potential to amplify the effects of DT, leading to quicker and more pronounced declines in their physical conditioning variables and adversely affecting their health [17].

DT in individuals with SCI can lead to changes in BC, metabolic profile, and variables related to the cardiorespiratory system [18,19]. The interruption of physical exercise impacts the increase in adipose tissue, decrease in muscle mass, alteration in metabolism, reduction in maximal oxygen consumption, glucose, and lipid imbalance [20]. Additionally, alterations in blood pressure and heart rate may manifest, raising the possibility of cardiovascular complications [21].

Although the phenomenon of DT has been studied in the general population, emphasizing the importance of regular physical exercise for health maintenance [9,16,22] in individuals with SCI, the literature is limited to observing the behavior of DT experienced by the injured limbs in exercise programs aimed at managing their condition [17,20].

In this context, the present study distinguishes itself as it investigates the impact of DT in relation to exercises targeting the active musculature, not affected by the injury, in individuals with SCI. This contrasts with the predominant approach, seeking to understand how long-term DT behaves in measures of physical performance, MH, and body parameters in this population.

This study emerges as a significant contribution to the scientific literature and for professionals who work directly with the training of individuals with SCI. By investigating the consequences of DT, this work seeks to fill a gap by addressing the long-term effects on active musculature, which is often marginalized in conventional research of this population. DT has been studied in a broader spectrum of the population without disabilities, primarily in athletes and the elderly population [9,15,16,23]. However, by focusing on individuals with SCI, this study recognizes and highlights the unique needs and responses of this population.

In this sense, the present study becomes relevant as the knowledge generated by it can assist health professionals in prescribing training for individuals with SCI. Additionally, in-depth knowledge of how DT affects physical performance variables, MH, and BC indicators in individuals with SCI could support more effective and personalized training programs, rehabilitation strategies, and health maintenance for this population.

Given the above, the aim of this study was to evaluate the effect of DT on physical and MH indicators in individuals with SCI, with a particular focus on MS, functional agility, MH, and BC.

## 2. Materials and Methods

This research is characterized as an observational study and a case series due to its intention to identify and record changes in health indicators after a period of DT without intervening in or modifying the training regimen of the participants [24].

The sample for the study was a convenience sample and consisted of five individuals with SCI with previous experience of one year in strength training, thoracic level injury, and of both sexes. The inclusion criteria were (A) having SCI at the thoracic level, (B) being physically fit to participate in physical tests, as determined by a medical examination, (C) possessing independence in performing activities of daily living, (D) not having musculoskeletal or cardiometabolic problems that limited or contraindicated the practice of planned physical tests, and (E) not participating in other regular physical exercise programs.

All evaluated individuals participated voluntarily, signed an informed consent form (ICF), and received information about the study, as determined in Resolution 466/2012 of the National Health Council. 

The study was approved by the Research Ethics Committee involving human beings of the Federal University of Viçosa, Minas Gerais, Brazil, and was conducted under license number CEAP: 51624715.2.0000.5153.

All study procedures were developed in the Strength Laboratory of the Department of Physical Education at the Federal University of Viçosa.

### 2.1. Detraining Protocol

The sample of the present study was part of a training program (Projeto Fortalecer) that was interrupted in December 2019. However, with the COVID-19 pandemic that affected Brazil and the world in 2020 and 2021, this interruption continued until September 2022. Thus, the study evaluated the effect of 33 months of DT on the investigated variables.

Prior to the DT period, the volunteers of this research participated in a 12-week resistance training program in which interventions occurred twice a week, each session lasting approximately 60 min. The volunteers performed 8 exercises targeting functional muscle groups, performing 3 to 4 sets of 8 to 12 repetitions for each exercise, with a rest interval of 45 s to 1 min between sets in the first two weeks, reducing to 30 s in subsequent weeks.

The training was designed to be performed entirely in the wheelchair, minimizing the need for adaptation. It included eight exercises: elbow flexion with shoulder extension and scapular adduction; shoulder abduction in the neutral arm position; elbow flexion with forearm supination; elbow extension with shoulder adduction (participants facing away from the apparatus); wrist flexion with forearm supination; wrist flexion with forearm pronation; elbow flexion and extension with shoulder abduction and adduction in a vertical pressing movement; and horizontal shoulder adduction with elbows in flexion.

The training load was controlled through the subjective perception of effort (SPE) [25], with intensity varying between 7 and 9 on the SPE using the OMNI-RES scale [25]. Intensity control was achieved by adjusting repetitions, sets, and training sessions, so when the individual’s SPE fell below the set level, the intensity would be adjusted to always be above 7 on the SPE.

To quantify and compare the total training load, the total mass moved in all eight exercises by all individuals was summed and multiplied by the weekly training volume for each of the 12 intervention weeks.

An undulatory periodization was adopted, characterized by greater variation in volume and intensity of the training session, providing frequent changes in stimuli, thereby stimulating the neuromuscular system’s adaptation to each training session and avoiding stagnation in strength gains [26].

The DT in this study occurred due to the interruption of the exercise program, which was prolonged due to the COVID-19 pandemic. During this interruption period, the participants were not monitored regarding the maintenance of their physical activities. Considering the context of the pandemic, it is plausible to believe that physical activity levels were restricted to performing essential daily life activities, given the restrictions and limitations imposed by the sanitary situation. Despite not accompanying them during the COVID-19 pandemic, at the end of it, immediately before returning to Fortalecer project activities, the participants reported that they had not participated in any physical exercise or physiotherapeutic program during the 33 months of interruption of project activities.

### 2.2. Procedures

To ascertain the effects of DT on the variables of interest, a 33-month interruption of the exercise program was observed. Assessment tests were conducted before and after the DT period to evaluate different manifestations of MS, functional agility, perception of MH status, and BC.

#### 2.2.1. Muscle Strength

To evaluate isometric strength, the maximum voluntary isometric contraction (MVIC) test of the upper limbs was used, employing a load cell or strain gauge (MK, model CSL/ZL-1T, Brazil) with a sampling frequency of 1000 Hz. The load cell was placed on CrossOver machine (Scorpions Fitness, Formiga, Brazil), such that one end was attached via a chain to the lower part of a stirrup handle and the other end fixed to the steel cable, which is pulled when the lever arm of the machine is moved. Before performing the test, the device was adjusted so that the elbow of the participants was at a 90° flexion angle. On the evaluator’s command, the participant performed a maximum isometric tension of the biceps brachii for 5 s. During execution, verbal encouragement was given to induce greater tension and maintain maximum levels throughout the test. Two attempts were made, separated by a 2-min interval, with the highest value obtained in the two attempts considered [27].

For the evaluation of dynamic strength, the one-repetition maximum (1RM) test was used through the elbow flexion exercise on CrossOver machine (Scorpions Fitness, Formiga, Brazil). The initial position was sitting with the back supported by the apparatus, one hand holding the side support of the chair, and the other holding a stirrup handle with the elbow extended to 0°. The volunteer was asked to flex the elbow to approximately 160° and return to the initial position. Before determining 1RM, a warm-up was performed consisting of 4 repetitions with a load of 50% of MVIC. After the warm-up, the volunteer’s effort perception was assessed using the OMNI-RES scale from 0 to 10 [25,28]. The load was increased based on the evaluator’s discretion, according to the ease of execution and the participant’s perceived effort, and the volunteer was asked to perform two repetitions with the new load. The load was increased until the participant could only perform one repetition. A maximum of 5 attempts were made to determine 1RM, with a 2-min rest interval between each attempt [27,29].

Upper limb power was also assessed using the same machine used for the 1RM evaluation. Three different loads were used for power assessment, derived from percentage values of 1RM (40%, 60%, and 80% of 1RM), where the participant was asked to perform the elbow flexion movement (concentric phase of the movement) as fast as possible. The return of the elbow to the initial position was controlled, with a 1 to 2-s micro-pause, to prevent the effect of accumulated elastic force from interfering in the subsequent execution. The loads for this test were randomized for each subject to control potential bias related to learning effects or cumulative fatigue action. In each load, three repetitions were performed with a 2-min rest interval between the loads, using the highest measure of the 3 repetitions [27].

A linear position transducer or encoder (Chronojump Boscosystem, Barcelona, Spain), with a sampling frequency of 1000 Hz, and the Chronojump software, version 1.6.2 (Chronojump Boscosystem, Barcelona, Spain), were used to determine power values. Through this instrument, information on average power (AP) and peak power (PP) was obtained.

#### 2.2.2. Functional State

To assess functional agility in wheelchairs, the adapted zigzag test (Texas fitness test) was performed [30]. The aim of the test was to traverse the total distance of a 6 × 9-m rectangle, requiring changes in direction, with the highest possible speed and efficiency. Each participant, using their own wheelchair, navigated the test course marked by five cones. On the evaluator’s signal, the participant propelled the chair through the course as quickly as possible. Five attempts were made, with a 5-min interval between them. The first attempt was for course recognition and was performed at a slow speed. The second was a high-speed recognition run. The following three attempts were considered valid for the test. A stopwatch with a hundredth of a second precision was used, and the result was the shortest time out of these three attempts.

#### 2.2.3. Perception of Mental State: Anxiety, Depression, and Mental Distress

To assess the presence of anxiety symptoms, the Beck Anxiety Inventory (BAI) was used, which consists of 21 items where the individual must indicate the severity level of the symptom on a four-point scale. The total score ranges from 0 to 63 and allows for the assessment of the intensity level of anxiety symptoms. The classification described in the manual recommends that the level of anxiety be classified as minimal (0–7), mild (8–15), moderate (16–25), or severe (26–63) [31].

For the evaluation of depression, the Beck Depression Inventory (BDI) was employed. This inventory consists of 21 items, including symptoms and attitudes, with intensity varying from 0 to 3. The items pertain to sadness, pessimism, feeling of failure, lack of satisfaction, guilt, sense of punishment, self-depreciation, self-accusations, suicidal thoughts, crying spells, irritability, social withdrawal, indecisiveness, body image distortion, work inhibition, sleep disturbance, fatigue, loss of appetite, weight loss, somatic concern, decreased libido [32,33].

For the assessment of mental disorders, the Self Report Questionnaire (SRQ) was used to evaluate the suspicion of psychiatric disorder symptoms through questions about the patient’s life. The Portuguese version of the SRQ 20 determines the cutoff point for not presenting psychotic morbidity as ≤8 for women and ≤6 for men [34].

#### 2.2.4. Body Composition

BC was assessed with a full-body dual-energy X-ray absorptiometry (DXA) scan (GE Healthcare Lunar Prodigy Advance DXA System, software version 13.31, Chicago, IL, USA). The equipment was calibrated prior to conducting the scan. The precision presented by a similar device [35] was 2.3% for total body mass (TBM), 1.6% for FM, 0.3% for LM, and less than 0.1% for bone mineral content (BMC).

Each volunteer’s evaluation was conducted after a 30-min wait and under the same conditions. During the DXA measurements, the volunteer lay supine on the device, with upper limbs extended and parallel to the trunk, hands pronated, and resting on the device. The lower limbs were also extended, with a standard separation at hip width, and secured by a strap holding the ankles. They were instructed to remain as still as possible for the duration of the scan [36,37]. Each scan took about seven minutes, and through them, an automatic calculation of the DXA outcome parameters (TBM, FM, LM, and BMC) was obtained.

### 2.3. Statistical Analysis

For the analysis of the data obtained in the present study, the Jacobson and Truax (JT) method was employed [38]. This statistical method has been used in research and interventions with single subjects or small samples [39]. The JT method is an alternative for research/interventions that lack a control group design and aims to investigate the reliability of change index (RCI) pre and post-intervention and the clinical significance (CS), which checks if the impacts observed in the last evaluation had an effect in the participant’s daily life, contributing to the application of these acquired behaviors in other environments [39].

For the calculations of the JT Method, the global score from the first evaluation (EV2019) of all participants was considered. To identify the cutoff point for CS, criterion A was used, which is indicated when normative data of the functional population are not available [38]. To understand the results regarding the RCI, the central diagonal line that separates the positive differences (post > pre) above and the negative differences (post < pre) below should be observed. There are also two dashed diagonals that delimit the area of uncertainty, indicating that participants located in this range had no reliable change (NC). These lines are drawn according to mathematical formulas based on the variability of the results (standard deviation, standard error, reliability of the instrument).

Regarding CS, the analysis of the graphs is performed through four quadrants formed by the crossing of the vertical and horizontal lines. To assert that there was a clinically significant change, participants must be situated in the quadrant above the horizontal line and to the left of the vertical line. The cutoff point calculation for CS was based on criterion A and is used when there are no normative population data, allowing for estimation of mean and standard deviation according to a dysfunctional sample.

In addition to the JT method, the effect size (ES) was calculated using Cohen’s d test to verify the magnitude of the result of the DT period on the analyzed variables, the standard error of measurement (SEM), and the minimal detectable difference (MDD), with the purpose of enhancing the analysis of the changes promoted by DT. The effect values were based on Cohen’s proposal, being classified as “small” (<0.2), “medium” (0.2 to 0.8), and “large” (>0.8) [40].

The calculation of the SEM was necessary to obtain the MDD, and then, the MDD with a 95% confidence level was verified. All calculations were performed in Microsoft^®^ Excel version 2010.

## 3. Results

The sample of the present study consisted of five individuals with chronic phase SCI with an average age of 46.2 ± 13.9 years, 60% of whom were women (n = 3). The injuries of all study participants were complete in the thoracic region. The causes of the injuries were varied (shooting, car accident, and fall), and the average duration of the injury was 19.6 ± 17.0 years.

Table 1 presents the descriptive analysis for each of the variables analyzed in the group of evaluated volunteers.

No participant was affected by the SARS-CoV-2 virus or reported any other infectious disease that could affect their health status during this study.

**Figure 1:**
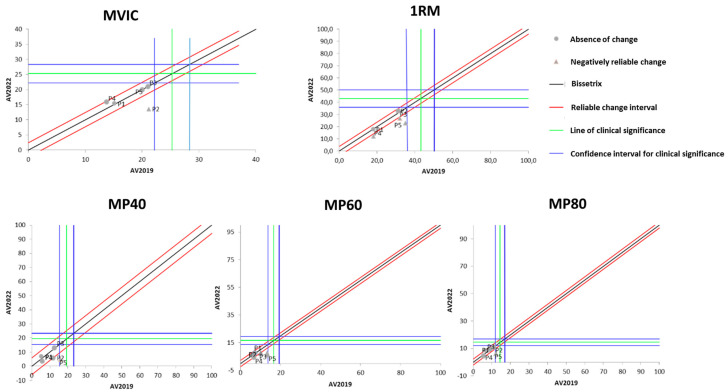
Dispersion of the differences between the EV2019 and EV2022 evaluations, measuring the effect of DT on the test results for assessing different manifestations of MS in individuals with SCI. Source: author.

Figure 1 presents the effects of DT that occurred between the two evaluations (2019 and 2022) on the results of isometric, dynamic, and power MS. It is observable that the DT period did not cause significant losses in maximum isometric strength in most of the study participants. On the other hand, three out of the five participants showed a significant reduction in maximum dynamic strength after the DT period.

Regarding muscle power (MP), Figure 1 demonstrates that there was no change in it after the DT period when measured at 40% of 1RM. When measured at 60% of 1RM, MP experienced a significant reduction in four of the five study participants. As for MP measured at 80% of 1RM, only one participant showed a significant reduction, while the others did not demonstrate changes with the DT period.

**Figure 2:**
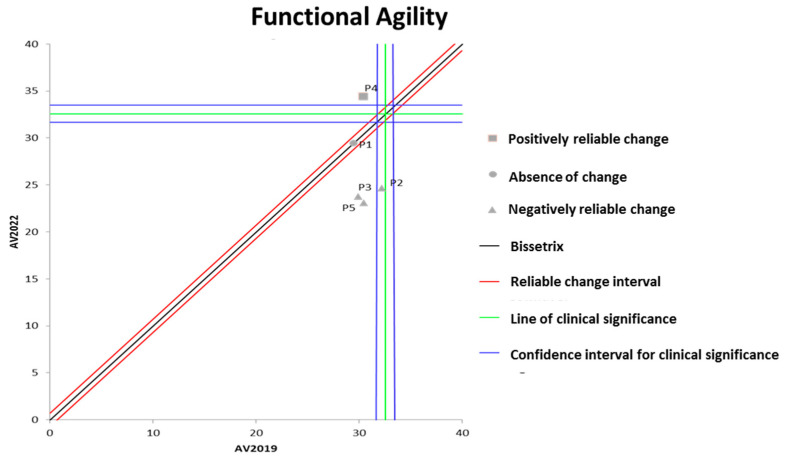
Dispersion of the differences between the EV2019 and EV2022 evaluations, measuring the effect of DT on the results of the zigzag test for assessing functional agility in individuals with SCI. Source: author.

Figure 2 shows the effects of DT that occurred between the two evaluations (2019 and 2022) on the results of functional agility assessed by the wheelchair zigzag test. It can be observed that the DT period resulted in significant losses in functional agility in three out of the five participants evaluated.

**Figure 3:**
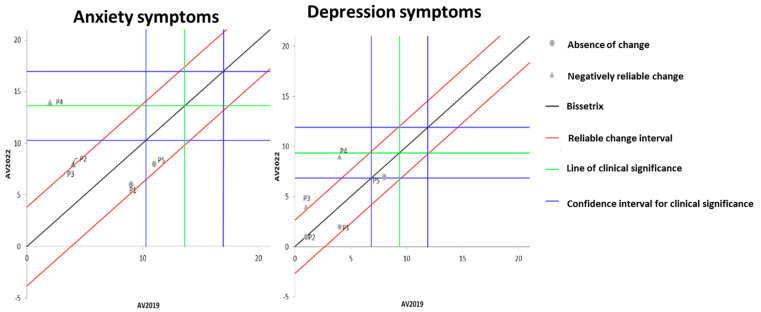
Dispersion of the differences between the EV2019 and EV2022 evaluations, measuring the effect of DT on the results of anxiety and depression for assessing MH in individuals with SCI. Source: author.

Figure 3 illustrates the effects of DT that occurred between the two evaluations (2019 and 2022) on the results of MH, assessed by anxiety and depression questionnaires. It is observable that the DT period led to a significant worsening of anxiety symptoms in three volunteers, while the other two showed no change. Regarding depression symptoms, two participants exhibited a significant worsening of symptoms, while the remaining three did not demonstrate any significant change.

**Figure 4:**
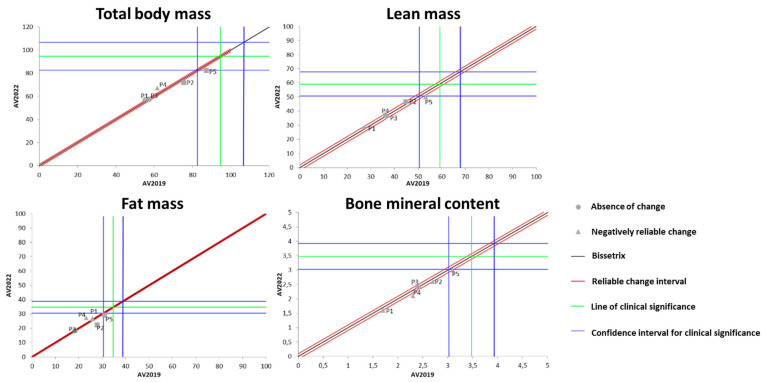
Dispersion of the differences between the EV2019 and EV2022 evaluations, measuring the effect of DT on the DXA results for assessing BC in individuals with SCI. Source: author.

Figure 4 shows the effects of DT that occurred between 2019 and 2022 on the results of BC measured by the DXA method. It was identified that the total body mass behaved heterogeneously during the DT period among the study participants (one increased, two remained unchanged, and two lost total body mass).

Regarding LM, four out of the five participants showed no changes due to the DT period, while one participant experienced a significant loss of LM during this time. For the variable of FM, a similar pattern to total body mass was observed, i.e., one participant increased FM, two remained unchanged, and two lost FM. Finally, for BMC, four out of the five evaluated individuals showed no significant change, while one individual exhibited a significant increase in this variable.

**Figure 5:**
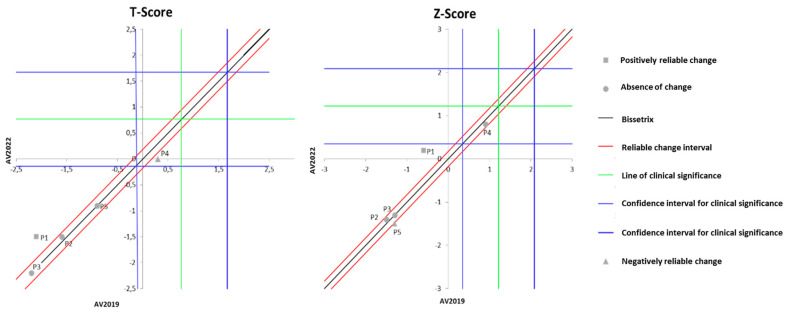
Dispersion of the differences between the EV2019 and EV2022 evaluations, measuring the effect of DT on the DXA results for assessing BMD in individuals with SCI. Source: author.

Figure 5 displays the effects of DT that occurred between the two evaluations (2019 and 2022) on the results of BMD. It can be observed that the DT period did not alter the T-score in four out of the five individuals with spinal cord injury (SCI) evaluated, although one participant exhibited a significant decline in T-score values. Similarly, the Z-score also remained unchanged in the majority of the evaluated individuals (three volunteers), while two participants showed distinct results: one with significant improvement and another with a decline.

## 4. Discussion

The present study investigated the long-term impacts of DT, over a period of 33 months, on measures of physical performance, MH, and BC parameters in individuals with SCI. The findings indicated a decline in various components such as MS, functional agility, anxiety, depression, and BC, demonstrating that DT has a considerable impact on the lives of this population.

MS plays a crucial role in the health and QoL of individuals with SCI [5]. The results of this study identified that isometric strength, the ability to generate muscle tension without movement, remained stable during the DT period. There appears to be a scarcity of studies that seek to identify the effects of DT on the strength of individuals with SCI, particularly regarding the evaluation of active musculature not affected by the injury. Thus, considering the changes caused by SCI, it can be compared to an accelerated model of aging [41].

The results corroborate the findings of Chen et al. (2018), where elderly women with sarcopenia maintained isometric strength derived from 8 weeks of kettlebell training, even after 4 weeks of DT [42]. Considering other populations, Araujo et al., (2021) found that healthy individuals and those with HIV/AIDS who underwent 15 weeks of concurrent training (strength and aerobic) and 5 weeks of DT maintained isometric strength without significant losses [43]. Although the results of these studies point in the same direction, it is important to note that the training methodology and especially the sample were different from the present investigation, as this study worked with individuals with SCI, while the cited studies investigated the elderly and patients with HIV/AIDS.

Contrary to these results, Häkkinen et al., (2022) investigated a group of healthy women for 10 weeks of resistance training followed by 5 weeks of DT and observed decreases in isometric strength −6.6 ± 3.6% (*p* < 0.01) [44]. Similarly, Gomez-Illan et al., (2020) investigated individuals with multiple sclerosis for 8 weeks in high-intensity resistance training, 90% 1RM, and reported losses in isometric strength after 10 weeks of DT [45].

It is important to highlight that the studies mentioned varied between 5 and 8 weeks of DT period. In contrast, the present investigation conducted a period of 33 months, aiming to fill a gap in the literature, as this is a long period of DT, thus adding originality to the work.

To explain these results regarding isometric strength, it should be considered that individuals with SCI benefit from the effects of training with a lower volume than individuals without SCI [46,47]. Therefore, it is believed that the movements of supporting and suspending one’s own body during moments of transfer and movement with the wheelchair serve as sufficient stimuli for the maintenance of isometric strength, given the need to support one’s own body weight in various activities such as bathing, moving from bed to chair and vice versa, among other tasks.

Upper limb MS is considered a relevant physical capacity for wheelchair propulsion and, therefore, for performing locomotion and transfer activities [48]. Furthermore, MS contributes to the prevention of secondary complications, such as pressure ulcers, respiratory, and postural problems [5,6,49].

In relation to dynamic strength, the capacity to generate muscle tension with movement, the results showed a decline in this variable. These findings are in line with the study by Schott, Johnen, and Holfelder (2019), which identified reductions in 10RM dynamic strength (*p* < 0.001 for all measures) in older adults after 6 weeks of DT following 26 weeks of resistance training with three sets of 10-12RM at intensities between 70 and 80% of 1RM [50]. Additionally, in a controlled randomized clinical trial in which 95 elderly women were randomly allocated to four experimental resistance training groups (strength, power, absolute strength, and relative strength) and one control group, undergoing 20 weeks of training with intensity controlled between 6 and 8 points of SPE and equalized volume between 25 and 30 repetitions for each group, Mazini Filho et al. (2022) observed, after 4 weeks of DT, significant losses in MS (*p* < 0.05) for all groups, even though strength values did not return to baseline [15].

Furthermore, two groups of healthy women, eccentric training vs. concentric training, subjected to 10 weeks of resistance training and undergoing 5 weeks of DT, showed declines in dynamic strength of −6.6 ± 3.6% (*p* < 0.01) for the eccentric group and −8.0 ± 4.5% (*p* < 0.001) for the concentric group in the 1RM bench press test [44]. Additionally, in a systematic review with older adults and middle-aged individuals, it was observed that when the DT period exceeded the duration of resistance training, the benefits of resistance training were not maintained in relation to MS [51]. Since the results indicated stability in LM and a reduction in dynamic strength, it is believed that the reduction in the ability to generate voluntary force is due to neural changes [52].

Perceiving the negative impact of DT on the analyzed variable, it is essential that individuals with SCI engage in specific training programs to maintain and improve MS. These programs can include progressive resistance exercises, strength training with weights or resistance machines, functional electrical stimulation, and other therapeutic interventions aimed at muscle strengthening [6,46].

The product of muscle force and movement velocity results in MP [53]. Regarding this variable, a reduction in MP at 60% of 1RM was observed. The behavior of MP observed during DT periods tends to show a decrease in performance levels, as observed by Mazini Filho et al., (2022) in their study with elderly women, where 4 weeks of DT were sufficient to reduce upper and lower limb MP (all *p* < 0.05) generated by 20 weeks of resistance training [15]. Another study in which elderly men underwent 12 weeks of resistance training followed by an equal DT period also observed reductions in MP (−5 to −15%, *p* ≤ 0.004) [54]. However, these reductions were not sufficient to return to pre-training baseline values in both studies [15,54].

Additionally, wheelchair basketball athletes who achieved better results in the MP test in the flat bench press exercise at ~50% of 1RM showed superior physical performance [55]. Thus, it is possible to consider that MP is essential for performing explosive movements and wheelchair maneuvers [5]. In this sense, resistance training focusing on MP can increase movement efficiency and functional independence in individuals with SCI [56]. On the other hand, DT, by reducing MP, negatively affects movement efficiency and functional independence in people with SCI.

One of the abilities directly linked to MP is agility, the ability to change direction rapidly, which is a crucial factor for the independence and mobility of individuals with SCI, allowing them to perform daily activities and move more easily [5]. The reduction of this capacity leads to mobility restrictions, preventing individuals with SCI from moving autonomously and freely [5].

Resistance circuit training (RCT) has proven to be effective in improving upper limb MP and functional capacity in individuals with SCI, as evidenced by a wheelchair-adapted agility test [5]. Furthermore, RCT is a low-cost and highly ecologically valid strategy that can be performed in the individual’s own wheelchair without the need for specific adaptations. By incorporating movements that mimic activities of daily living and training stations that require agility and rapid changes of direction, RCT also stands out as a practical approach to improving functional agility [5].

During the training interruption period, there was a loss of functional agility measured by the adapted wheelchair agility test. Studies that conducted wheelchair agility tests were associated with the evaluation of this variable for the functional classification of wheelchair basketball athletes [57,58] and wheelchair handball [59]. This makes it difficult to compare the results found within this population. Therefore, when investigating the effect of DT on functional agility in different populations, the following can be found: Leitão et al., (2022) observed that 1 year of DT led to a loss of functional agility (−4.24% *p* < 0.01) in hypertensive elderly women, stemming from 9 months of multicomponent training [14]. The same authors also identified losses in agility (*p* < 0.05) after 3 months of DT in elderly women with high cholesterol and triglycerides [60]. In line with these findings, Park and Lee (2015) observed that 8 weeks of DT led to a significant loss of agility (*p* < 0.01) in elderly individuals with type 2 diabetes, although the results still remained high compared to the pre-training baseline [61]. Similar to this result, Blasco-Lafarga et al., (2020) found that the agility of elderly individuals after 7.5 months of DT was the variable that, despite showing losses, exhibited greater retention compared to other physical components (*p* < 0.001) [62].

As agility demands factors such as strength and speed, it is directly associated with anaerobic power and functional physical performance [63]. In this sense, the loss of agility in most participants may perhaps be explained by the alteration from fast-twitch white type II fibers to slow-twitch red type I fibers [5] due to a possible reduction in physical activity levels and lack of anaerobic stimuli caused by the interruption of resistance training during the COVID-19 pandemic quarantine.

The present study showed a worsening of MH in individuals, both for anxiety and depression, during the DT period. This phenomenon is evident in different investigated groups, namely elderly women with type 2 diabetes [64], badminton athletes [65], individuals affected by ischemic stroke [66], and patients diagnosed with Parkinson’s disease [67], revealing an increase in depressive indicators following periods of DT, with durations ranging from 8 weeks to 1 year.

It is worth noting that the methodological discrepancies in the studies presented in the previous paragraph reflect the diversity of approaches in studying DT in different populations and conditions, such as training intensity and type, DT exposure time, and assessment instruments. In summary, while some studies indicate an improvement in anxiety and depression symptoms with training, the sustainability of these benefits after DT periods appears to vary, with some studies indicating a partial reversal of psychological benefits and others not finding significant changes.

To explain the results of this study, it is interesting to highlight that there seem to be two effects affecting the MH status of the sample in this study. In addition to the effect of DT itself, one must consider the effects of the COVID-19 pandemic, which, according to systematic review studies [68,69], increased anxiety symptoms (6.33% to 50.90%) and depression symptoms (14.6% to 48.3%) in the general population in various countries due to the social isolation protocols adopted to combat the pandemic.

BC is a determinant factor in the health and rehabilitation of individuals with SCI, as changes in FM and LM can directly influence functionality, the risk of secondary diseases, and QoL [70,71,72]. High FM is associated with an increased risk of cardiovascular complications, while adequate LM is crucial for maintaining MS and preventing atrophy, being essential for daily activities and independence [6,73,74].

The results of the present study show that there was no change in LM during the DT period. In this regard, it is worth noting some methodological limitations, such as the lack of monitoring of physical activity levels, dietary control, and pharmacological control of the subjects in the sample during the analyzed period. Comparing the effect of DT on LM in other populations, this variable tends to decrease in women survivors of breast cancer [75], elderly women [76], and elderly men with osteosarcopenia [77]. Furthermore, according to a systematic review by Del Vecchio et al. (2020), the literature suggests that BC tends to result in a reduction in LM and an increase in body fat accumulation after DT periods [78]. In line with this statement, an increase in visceral adipose tissue was observed in individuals with SCI who underwent DT periods with monitored physical activity levels [20].

As individuals with SCI benefit from the effects of physical activity with lower volumes, it is believed that the activities performed in daily life may have been sufficient to maintain LM in most subjects in the present study [47,79].

BMD, which results from a dynamic process of bone tissue formation and resorption, and cross-sectional muscle area (CSA) are other aspects that deserve attention, as they are important indicators of bone health [80]. These aspects are often compromised in this population, leading to osteoporosis and a high risk of fractures [3,81].

The results found in this study did not observe changes in bone health variables during the DT period. However, individuals with SCI who underwent a period of 12 months of training followed by 12 months of DT showed a loss of the benefits generated by training in both BMD and CSA [18]. In line with the results of the present study, Gorgey et al., (2021) did not observe changes in BMD after 16 weeks of DT in individuals with SCI [20]. It is worth noting that the results of the compared studies correspond to an analysis of the limbs affected by the injury through studies involving electrical stimulation.

Physical exercise promotes osteogenesis and increases BMD and CSA by imposing mechanical loads on the bones through muscle contractions [82]. Therefore, the lack of movement in the affected musculature can lead to faster absorption of the bone content acquired through training compared to the still-active limbs, which is the case in the present study’s sample.

Analyzing the changes in LM, FM, BMD, and CSA, it can be considered that the evaluation and monitoring of BC are essential components of clinical management in individuals with SCI, aiming to optimize the results of intervention programs and promote a healthier lifestyle [71].

The present study has limitations that should be considered when interpreting its results. The use of a small group of participants restricts the generalization of the findings to the broader population of individuals with SCI. The lack of monitoring of participants’ physical activity levels may have influenced the results of BC and functionality. Additionally, the absence of comparative data with pre-training baseline data hinders a clear analysis of the participants’ physical condition evolution or regression in response to DT. To strengthen the quality of evidence in future studies, it is essential to include a larger number of participants, adequate monitoring of physical activity, a more homogeneous group with detailed classifications of SCI, and comparisons with baseline data.

Finally, as a practical implication to the clinical practice, we believe the maintenance of physical exercise practice in people with SCI, especially RT, is an efficient strategy for preserving or increasing muscle strength. This highlight is especially important because the improvement of muscle strength is related to better scores of functionality and independence in ADL in people with SCI. With these data in hand, professionals involved in rehabilitation and exercise programs can prescribe training protocols using RT to improve muscle strength manifestations and achieve benefits from an active lifestyle. These findings of the present study may also guide future rehabilitation protocols in public health.

## 5. Conclusions

This study highlights the negative consequences of prolonged DT in individuals with SCI, demonstrating reductions in dynamic MS, functional agility, and MH despite the preservation of isometric strength and BC. The stability of isometric strength suggests that daily activities may provide sufficient stimuli for its preservation, while the deterioration of MH underscores the importance of physical exercise for psychological well-being, especially during periods of isolation such as those imposed by the COVID-19 pandemic.

The findings reinforce the need for continuous and tailored training programs for individuals with SCI to improve functionality and MH and prevent secondary complications. The study’s limitations, including the sample size and lack of physical activity monitoring, point to the need for more detailed future research to optimize intervention strategies for this population.

## Figures and Tables

**Table 1:** Descriptive analysis of variables assessed to verify the effect of DT between 2019 and 2022 in individuals with SCI.

	EV2019					EV2022				
	Mean	SD	Median	Minimum	Maximum	Mean	SD	Median	Minimum	Maximum
MVIC (Kg)	16.70	5.03	17.75	8.80	21.20	19.00	6.14	16.00	13.60	28.80
1RM (Kg)	24.50	9.22	24.50	13.00	35.00	22.60	8.08	23.00	12.00	33.00
MP40 (Watts)	89.24	51.59	86.86	26.18	159.35	69.38	38.57	59.18	35.76	134.32
MP60 (Watts)	88.12	42.17	85.48	26.07	134.45	66.24	13.19	74.62	44.67	74.87
MP80 (Watts)	77.35	37.78	77.29	20.98	122.03	80.55	26.31	86.43	45.63	111.69
Zigzag (s)	32.55	5.17	30.41	29.45	42.93	27.10	4.81	24.68	23.10	34.46
Anxiety	5.40	4.39	4.00	1.00	11.00	8.50	4.12	8.00	4.00	14.00
Depression	6.00	3.39	8.00	1.00	9.00	4.75	3.86	4.50	1.00	9.00
TBM (Kg)	63.57	15.22	59.50	45.00	87.20	67.30	10.48	67.10	57.50	82.40
LM (Kg)	37.53	10.14	36.29	27.23	53.22	39.87	8.76	37.54	28.11	50.08
FM (kg)	23.77	56.00	24.58	16.28	30.93	24.86	4.42	26.81	18.35	29.31
BMC (Kg)	2.28	0.59	2.36	1.47	3.09	2.36	0.52	2.37	1.64	3.03
T-score (sd)	−1.50	1.04	−1.85	−2.50	0.30	−1.22	0.82	−1.50	−2.20	0.00
Z-score (sd)	−0.82	0.90	−1.20	−1.50	0.90	−0.64	1.06	−1.30	−1.50	0.80

EV2019—evaluation in 2019; EV2022—evaluation in 2022; SD—standard deviation; MVIC—maximum voluntary isometric contraction; 1RM—maximum voluntary dynamic contraction; MP40—muscle power at 40% of 1RM; MP60—muscle power at 60% of 1RM; MP80—muscle power at 80% of 1RM; TBM—total body mass; LM—lean mass; FM—fat mass; BMC—bone mineral content. Source: author.

## Data Availability

The raw data supporting the conclusions of this article will be made available by the authors on request.
